# Risk and Resilience Factors Impacting Treatment Compliance and Functional Impairment among Adolescents Participating in an Outpatient Interdisciplinary Pediatric Chronic Pain Management Program

**DOI:** 10.3390/children7110247

**Published:** 2020-11-22

**Authors:** Richa Aggarwal Dutta, Katherine S. Salamon

**Affiliations:** 1 Children’s Hospital of Philadelphia, Philadelphia, PA 19104, USA; 2 Nemours/A.I. DuPont Hospital for Children, Wilmington, DE 19803, USA; Katherine.salamon@nemours.org

**Keywords:** chronic pain, adolescent, risk, resilience, treatment compliance

## Abstract

Recurrent pain is a common experience in childhood and adolescence and can result in significant disability in youth, including poor quality of life, school absences, and reduced social activities. Evidence has linked adolescent risk and resilience factors with treatment outcomes. However, less research has focused on examining risk and resilience factors that may influence or predict adolescents’ compliance to treatment within an interdisciplinary pediatric chronic pain management program. Participants included 64 adolescents (*M* = 15.00 ± 1.69 years); 85.9% female, 84.4% Caucasian who presented to an initial evaluation in an interdisciplinary pediatric pain management program with their caregiver. Youth completed a series of questionnaires at the initial evaluation targeting pain acceptance, self-efficacy, pain catastrophizing, parental responses, pain intensity, and functional disability. Treatment compliance was measured at 3 and 6 months post-intake. Findings indicated that higher levels of adolescent-reported self-efficacy predict decreased treatment session attendance, whereas lower levels of acceptance and parental encouragement/monitoring of symptoms predict increased treatment compliance overall. Several adolescent-reported risk factors were associated with increased functional impairment among this sample. Results highlight the unique importance of risk and resilience factors within the developmental context of adolescence, while also emphasizing the need for further investigation of other relevant influences towards treatment compliance and functional impairment.

## 1. Introduction

Pediatric chronic pain (i.e., recurrent and persistent pain lasting longer than 3 months) is an increasingly common developmental health problem among youth [1,2]. Youth with chronic pain experience daily pain interference, including missed school, social skills difficulties, and increased family-related psychosocial stressors [3,4,5]. They also report elevated levels of anxiety and depression and are at risk for continued chronic pain, other physical symptoms, and psychological problems into adulthood [6,7,8]. Although prevalence rates for chronic pain vary across the extant literature, research has reported median rates of 11–38% among children and adolescents [9]. It is especially important to consider the experience of pediatric chronic pain among adolescents, given that chronic pain and pain-related disability peak during this developmental period [10]. The increase in chronic pain during adolescence may be related to multiple factors that occur during this developmental stage and with puberty, including physical, cognitive, emotional, and social changes. Evidence specifically indicates that headache and migraine [11] and abdominal and musculoskeletal pain [12] frequency increases with age and incidence rates peak at 14 to 18 years of age. As such, it is particularly important to conceptualize the adolescent’s experience of pain, and also to discern whether targeting various individual factors often involved in the development and maintenance of pain and functional disability [1,13] could lead to more effective treatment.

Evidence suggests that early interdisciplinary interventions (i.e., a combination of medical, psychological, and physical approaches) can help prevent or attenuate sequelae associated with chronic pain [14]. Among psychological interventions for youth with chronic pain, cognitive-behavioral therapy (CBT) is the most widely used and well-researched [15,16]. CBT for pain management is a type of therapy that focuses on altering negative cognitions related to pain and increasing a focus on healthy lifestyle habits and managing behaviors that occur secondary to pain (e.g., decreasing sedentary behaviors). Despite evidence of the efficacy of CBT for pediatric chronic pain, many adolescents and families do not have access to this treatment or, for a variety of reasons, do not comply with treatment recommendations [15,17,18]. Adolescents’ active commitment to care is emphasized within the construct of compliance, which can more specifically be viewed as their attendance of treatment sessions as well as their use/engagement in treatment recommendations across sessions [18]. It is important to better understand how adolescents engage in prescribed treatments, as well as potential facilitating or mitigating factors that influence behavior relevant to treatment given the impact of chronic pain and implications for the future.

Certain risk factors associated with the experience of pediatric chronic pain that can exacerbate symptoms have been identified [19]. Pain catastrophizing is thought to serve as a means for communicating pain-related distress and may result in heightened assistance and empathic responses from parents [20,21]. Research suggests that the extent to which adolescents catastrophize about pain is impacted by reinforcement (e.g., protective parental responses) and, in turn, is positively correlated with functional disability [22,23]. Pain catastrophizing has been shown to mediate the relationship between parent protectiveness and functional disability among adolescents with chronic pain at the time of an initial pain clinic evaluation [22] and again over a two-month time period [10]. Finally, evidence also suggests that co-occurring anxiety puts youth with pediatric chronic pain at higher risk for more severe and/or frequent negative outcomes, including increased functional disability and pain symptoms [24].

Stable individual factors that appear to serve as resilience resources in the context of chronic pain have also been identified [25] and may play a role in improved treatment compliance and decreased functional impairment among adolescents. In the context of chronic pain, resilience is best defined as protective factors and healthy responses to pain symptoms that may minimize dysfunction, help with adaptation to pain, and/or promote well-being and growth [25]. Pain acceptance has recently gained increasing attention and empirical support as an important predictor of functional outcomes among youth with chronic pain [26]. Pain acceptance is defined as an individual’s willingness to live life with pain, without efforts to control or avoid pain, and to pursue a life consistent with personal values [27]. Preliminary evidence also suggests that higher pain acceptance is concurrently associated with better functioning among youth with chronic pain [28]. Increased pain acceptance in the context of interdisciplinary chronic pain rehabilitation predicts reductions in depressive symptoms, pain catastrophizing, and functional disability among children and adolescents [29].

Pain self-efficacy also serves as a resilience factor that influences pain management by denoting a confidence in the ability to function while experiencing chronic pain [30]. Pain self-efficacy has been linked with improved emotional and physical functioning, as well as higher levels of self-esteem [30]. Self-efficacy emerged as a partial mediator of the relation between pain-related fear and disability and pain-related fear and school functioning in a group of youth with chronic headaches [31]. Additionally, evidence has indicated that pain self-efficacy in conjunction with pain acceptance were linked with functioning improvements, such as increased school attendance and less depressive symptoms in a sample of youth with chronic headaches, after controlling for demographic and pain-related variables [32]. Taken together, high pain acceptance and high pain self-efficacy can be viewed as robust indicators of resilience in the context of chronic pain.

Although previous research has investigated adolescent risk and resilience factors associated with chronic pain, relations among these factors with treatment compliance and functional impairment are still poorly understood from a developmental perspective. Adolescents with chronic pain should be viewed as individuals who are passing through a critical developmental stage while also dealing with achieving autonomy and self-management within the context of a chronic illness [9]. In addition, adolescents are more likely to follow through with therapy recommendations when they believe that the treatment will be effective [17]. A better understanding of the factors that contribute to adequate compliance could allow for improved recommendations at the onset of treatment in an effort to improve follow through and, ultimately, decrease the disability associated with chronic pain.

The current study aimed to examine risk and resilience factors that may influence or predict adolescents’ compliance to treatment within an interdisciplinary pediatric chronic pain management program. Firstly, this study evaluated whether and to what extent risk factors (i.e., pain catastrophizing, maladaptive caregiver responses to symptoms) and resilience factors (i.e., high acceptance of pain, high pain self-efficacy, adaptive caregiver responses to symptoms) may influence treatment compliance among adolescents experiencing chronic pain (i.e., 3 months and 6 months post-treatment after an initial evaluation). Secondly, the study evaluated whether and to what extent risk and resilience factors were concurrently associated with functional impairment variables among adolescents with chronic pain. Given the evidence that co-occurring anxiety places youth with chronic pain at higher risk for negative outcomes [24], this variable was used as a co-variate in all analyses to control for other potential factors that may contribute to increased functional disability and/or poor treatment compliance among the sample. It was hypothesized that after controlling for self-reported adolescent anxiety, the following factors would be significantly associated with higher levels of treatment compliance and lower levels of functional impairment: (1) increased acceptance of pain; (2) adaptive caregivers’ responses to adolescents’ pain symptoms (i.e., decreased protectiveness; increased encouragement/monitoring); (3) decreased adolescent catastrophic thoughts and feelings about pain; and (4) increased perceived self-efficacy for functioning when in pain.

## 2. Materials and Methods

### 2.1. Participants

Participants included 64 adolescents diagnosed with chronic pain who presented with their caregiver to their initial visit to an interdisciplinary pediatric chronic pain management program within a children’s hospital located in the northeastern region of the United States between November 2015–March 2017. The adolescents participating in this study were representative of patients currently seen in the interdisciplinary pain clinic, which include youth ages 11 to 18 years old that are predominantly female and non-Hispanic Caucasians. Consent and assent forms were collected from the caregivers and adolescents for every participating family before enrollment. Adolescents were eligible for participation if they (a) contacted the interdisciplinary pediatric chronic pain management program, (b) arrived at their initial evaluation, (c) were English speaking, and (d) had written parent/guardian consent and youth assent. No participant was excluded based on sex or racial/ethnic origin. All subjects gave their informed consent for inclusion before they participated in the study. The study was conducted in accordance with the Declaration of Helsinki, and the protocol was approved by the Institutional Review Board at Nemours/A.I. DuPont Hospital for Children. The board reference number is 754,273 and the most recent continuing review was approved on 4 January 2020.

Data from the present study were drawn from assessments when youth arrived for their initial intake (Time 1), and compliance was evaluated at 3 months (Time 2) and 6 months (Time 3) post-intake if they were recommended to return for treatment. At Time 1, participants were 64 youth ranging from 11–19 years (*M* = 15.00 ± 1.69 years; 85.9% female; 84.4% Caucasian, 6.3% African American/Black, 1.6% Hispanic/Latino, 1.6% Asian, 4.7% Mixed Race, and 1.6% “Other”). Of the families which consented, two were not recommended to return for treatment after their initial intake, due to distance, as the pain clinic serves a wide catchment area. Given that these youth were still referred to the clinic, their data was still included in the analyses in order to understand the patient population and the concurrent relationship between individual factors and functional impairment variables at Time 1 However, these data were not included in compliance analyses at Time 2 and Time 3, since these youth did not receive treatment. In addition, one family rescinded their consent to participate in the study prior to their 6-month assessment. Therefore, Time 2 (*n =* 62) and Time 3 (*n* = 61) included fewer participants than Time 1, though the low levels of attrition precluded any additional analyses for participants with complete verses incomplete data.

### 2.2. Procedure

The current study was approved by the Institutional Review Board of the affiliated institution where participants were recruited. Families who contacted the interdisciplinary pain program were consented and assented to participate in the study once they arrived for their initial visit. Each youth that enters the program receives, as standard care, an evaluation from a medical provider, a psychologist, and a physical or occupational therapist. These families then receive treatment recommendations, including (but not limited to) individual, family, and group psychotherapy (i.e., 6–10 sessions of CBT for pain and stress management); medication(s); physical/occupational therapy; and access to alternative treatments (e.g., yoga, massage, and healing touch). Families had the opportunity to decline treatment recommendations and discontinue involvement with the program. As previously mentioned, 96.9% of families which consented (*n* = 62) were recommended to return for treatment following their initial intake (Time 1); they were re-assessed at 3 months (Time 2) and 6 months (Time 3) for treatment compliance. These time points were selected based on the typical length of psychological treatment (e.g., CBT) within the clinic (6–10 sessions), as well as the fact that most youth continue to attend treatment sessions for an even longer period of time for symptom management. No data were excluded from analyses based on family’s follow-through with treatment recommendations provided during the initial evaluation within the pain program. Specifically, all families that were recommended to return for CBT were included in analyses (whether or not they followed up with participating in treatment) in order to understand the patient population and the concurrent relationship between individual factors and functional impairment variables at Time 1. However, these data were not included in compliance analyses at Time 2 and Time 3 since these youth did not receive treatment.

During the initial evaluation (Time 1), adolescents and their accompanying caregiver were approached in the waiting room to provide assent and consent, respectively, to participate in a study and families were informed that their participation could aid others in developing a better understanding of chronic pain among youth. It was explained that participation in completing the questionnaires was not related to treatment recommendations or care provided by the clinical team. Adolescents were asked to complete measures about demographics, anxiety symptoms, their caregivers’ responses to their pain symptoms, their own catastrophic thoughts and feelings about pain, perceived ability to function when in pain, and level of acceptance of pain. Functional impairment variables (i.e., functional disability, self-reported pain intensity) were also assessed at the initial intake (Time 1). Following the initial evaluation, families who were advised to return for brief CBT for pain management (6–10 sessions) were formally included in the study to track their recommended follow-ups for treatment. If these families returned for follow-up treatment sessions, they were assessed for compliance at 3 months (Time 2) and 6 months (Time 3) after the initial intake. Treatment compliance was examined at Time 2 and Time 3 by conducting comprehensive chart reviews to assess whether youth returned for treatment (i.e., number of sessions cancelled, number of no-show sessions); number of sessions attended; and level of self-reported use and/or engagement with treatment recommendations.

### 2.3. Measures

The following measures were administered to adolescents over the course of the study, as detailed below.

Demographics and Pain Ratings. At Time 1, adolescents were asked to complete a demographic questionnaire. This form asked adolescents to report on basic demographic information related to themselves (i.e., age, gender, ethnicity).

Adult’s Responses to Children’s Symptoms—Child form (ARCS). The ARCS-Child form [33,34] assesses the youth’s perception of their caregivers’ responses to pain symptoms. It contains three factor-analytically derived subscales: Protectiveness (15 items; parental encouragement of illness behavior and putting the child in a sick role), Encouragement/Monitoring (8 items; parental responses that serve to monitor, reassure, distract, and encourage), and Minimization (6 items; parental responses that criticize illness behavior). At Time 1, adolescents rated each item on a 0 (never) to 4 (always) scale. The Protectiveness (α = 0.88) and Encouragement/Monitoring (α = 0.84) subscales were used for the present study, with higher scores on each subscale indicating higher levels of protectiveness (considered a maladaptive response) and higher levels of encouragement/monitoring (considered an adaptive response), respectively.

Pain Catastrophizing Scale-Child (PCS-C). The PCS-C [35] is a 13-item measure that assesses different catastrophic thoughts and feelings that youth may experience when they are in pain. This measure is the child self-report version of the PCS that was administered in the parent-report form. At Time 1, adolescents rated how frequently they experienced each of the thoughts and feelings when they were in pain using a scale from 0 (not at all) to 4 (extremely). The PCS-C total score was used in the present study; scores can range from 0 to 52 (α = 0.80) with higher scores indicating higher levels of catastrophizing of symptoms (considered a risk factor).

The Chronic Pain Acceptance Questionnaire-Adolescent form (CPAQ-A). The CPAQ-A [28] is the adolescent version of the original CPAQ, a 34-item inventory designed to measure the acceptance of pain [36]. The CPAQ-A is a 20-item measure and includes two components of acceptance of chronic pain: (1) activity engagement (i.e., the level of participation in normal daily activities in the presence of pain) and (2) pain willingness (i.e., the relative absence of attempts to avoid pain). Each item is rated on a scale from 0 (never true) to 4 (always true). The total CPAQ-A score was used for the current study (α = 0.55) at Time 1 with higher scores indicating higher levels of acceptance of symptoms (considered a resilience factor).

Pain Self-Efficacy Scale (PSES). The PSES [30] is a 7-item measure that assesses self-efficacy for functioning when in pain. Example items include ‘‘How sure are you that you can take care of yourself when you have pain?’’ and ‘‘How sure are you that you can do well in school when you have pain?’’ Items are scored on a scale from 1 (very sure) to 5 (very unsure), with higher scores indicating higher levels of self-efficacy (considered a resilience factor). The total PSES score was used for the current study (α = 0.89) and administered at Time 1.

PROMIS Youth Pain Interference Short Form (PROMIS-YPI SF). The PROMIS-YPI SF [37,38] is an 8-item form derived from the item bank for Pain Interference developed by the National Institute of Mental Health-sponsored Patient Reported Outcomes Measurement Information System (PROMIS). This measure assesses youth’s perception of pain-related functional impairment within the past week. At Time 1, adolescents rated how often pain interfered with a variety of domains, including physical functioning, sleep, school performance, and emotional functioning, on a scale from 0 (never) to 3 (often) with higher scores indicating higher levels of functional disability. Raw scores were converted to T–scores (*M* = 50, *SD* = 10) using a standardized scoring template. This measure was used to index self-reported total functional disability for adolescents at Time 1 (α = 0.73).

PROMIS Youth Anxiety Short Form (PROMIS-YA SF). The PROMIS-YA SF [37,39] is an 8-item form derived from the item bank for Pediatric Anxiety developed by the NIMH-sponsored PROMIS. This measure assesses fear (e.g., fearfulness), anxious misery (e.g., worry), and hyperarousal (e.g., nervousness) over the past week. At Time 1, adolescents rated how often they experienced feelings in a variety of contexts, including home, school, and social activities on a scale from 0 (never) to 4 (always) with higher scores indicating higher levels of anxiety. The measure was used to index total self-reported anxiety among adolescents at Time 1, and was used as a control in all analyses (α = 0.92).

### 2.4. Functional Impairment

Functional impairment indices (i.e., functional disability, self-reported pain intensity) were assessed at Time 1 by using the following indices: (1) functional disability from the PROMIS-YPI SF, (2) self-reported highest/worst pain intensity ratings on a scale of 0 to 10 at time of assessment, and (3) self-reported pain intensity difference ratings (calculated by subtracting lowest from highest pain rating). Of note, participants were asked to rate their highest and lowest pain ratings on average for specific pain location.

### 2.5. Treatment Compliance

Treatment compliance was examined at each time point by conducting comprehensive chart reviews to assess number of cancelled treatment sessions, number of no-show treatment sessions, number of total sessions attended, and clinician-reported use and/or engagement with treatment recommendations (dichotomized in analyses as 0 = “no” and 1 = “yes” for each treatment session, then calculated as the percentage of the total number of sessions where recommendations were appropriately followed). Specifically, each session note was reviewed to track whether coping strategies introduced and taught at the previous session (e.g., diaphragmatic breathing, activity pacing, and thought challenging) had been actively practiced and utilized between sessions per the clinician’s report. Participants were specifically rated on whether they completed their homework assignment and expressed perceived benefit from skills that were introduced in the previous session. Although no formal guidelines were utilized to review patient charts, independent evaluators (i.e., doctoral and postdoctoral psychology trainees) rated participants using template session notes that specifically described such details.

### 2.6. Statistical Analyses

Descriptive statistics and bivariate correlations were conducted using SPSS Version 24.0 to examine relations among the predictor and outcome variables, as well as to explore distributions (e.g., low base rates, outliers, skewness, and kurtosis) and multicollinearity among variables that could unduly influence analyses. No transformations were performed and no outliers were removed after examining the distributions of variables. For subscales with <50% missing items, pro-rated raw scores were calculated using “response pattern scoring” to approximate values as if all items had been answered [40]. For example, if a participant answered 5 of 8 questions and answered these 5 items with the second lowest response option (2), all responses would be summed (2 + 2 + 2 + 2 + 2 = 10), then multiplied by the number of items in the subscale (8), and finally divided by the number of items that were answered (5). Prior to inclusion in multiple regression analyses, all variables were standardized (*z*-scored). Because of low base rates of no-show treatment sessions among participants, cancelled and no-show treatment sessions were combined into a “total missed treatment sessions” variable for analyses. Pain intensity difference ratings were calculated by subtracting the lowest from the highest self-reported pain ratings.

Primary analyses were conducted using Mplus Version 7.11 [41]. To address additional missing data not accounted for prorating scores, Mplus uses Full Information Maximum Likelihood (FIML) estimation, which conducts parameter estimation and estimates standard errors using all available data [42]. Given that other strategies for managing missing data (e.g., listwise or pairwise deletion, mean imputation) may bias an analytic sample [42,43,44], FIML permits inclusion of participants with missing data.

## 3. Results

### 3.1. Descriptive Statistics

Descriptive statistics are included in Table 1. Correlations among variables derived from separate measures were generally small in magnitude, whereas correlations among subscales drawn from the same instrument were moderate overall. Given that self-reported anxiety was correlated with key predictor and outcome variables in the study (i.e., pain acceptance and pain-related functional disability), it was used as a co-variate across analyses (Table 1).

### 3.2. Adolescent Factors with Treatment Compliance

Regression analyses considered prediction to each of the Time 2 and Time 3 treatment compliance variables from Time 1 self-reported adolescent risk and resilience factors, while co-varying Time 1 adolescent-reported anxiety (see Table 2). Results indicated (1) higher levels of Time 1 PSES adolescent-reported self-efficacy predicted the total number of missed sessions (i.e., number cancelled and/or no-showed) at Time 2; (2) lower levels of adolescent-reported acceptance on the CPAQ and lower levels of ARCS-A encouragement/monitoring both predicted the level of adolescent-reported use and/or engagement with treatment recommendations at Time 2; and (3) lower levels of Time 1 adolescent-reported acceptance levels on the CPAQ predicted the number of treatment sessions attended at Time 3. Adolescent factors did not predict the number of treatment sessions attended at Time 2, the number of total missed sessions at Times 2 and 3, or level of adolescent-reported use and/or engagement with treatment recommendations at Time 3.

### 3.3. Adolescent Factors with Functional Impairment

These analyses considered cross-sectional predictions to each of the Time 1 functional impairment variables from Time 1 self-reported adolescent risk and resilience factors, while co-varying Time 1 adolescent-reported anxiety (see Table 3). Results indicated the following: (1) lower levels of pain acceptance on the CPAQ were associated with increased adolescent-reported functional disability levels on the PROMIS-YPI SF; (2) lower levels of ARCS-A encouragement/monitoring and higher levels of ARCS-A protectiveness were associated with higher levels of adolescent-reported pain intensity; and (3) higher levels of ARCS-A protectiveness were associated with the pain intensity difference scores.

## 4. Discussion

This study explored risk and resilience factors contributing to treatment compliance and functional impairment among youth with chronic pain during adolescence. The investigation of adolescent-specific variables to confer resilience specifically has not been pursued to date. Contrary to initial hypotheses, there is some indication that adolescent-level resilience factors are associated with poorer treatment compliance. Specifically, higher levels of Time 1 adolescent-reported self-efficacy predicted an increased number of missed treatment sessions, whereas it was initially hypothesized that it would predict improved session attendance. Similarly, there was an inverse relationship between other resilience factors (pain acceptance and parental encouragement/monitoring of symptoms) and the number of treatment sessions attended and engagement in recommendations, whereas it was initially hypothesized that higher levels of such resilience factors would predict improved treatment compliance overall. Specifically, lower pain acceptance and lower levels of parental encouragement/monitoring of symptoms predicted more treatment sessions attended and higher engagement in treatment recommendations. Overall, these results suggest that adolescent resilience factors (i.e., high pain self-efficacy, high pain acceptance, adaptive caregiver responses to pain) may make adolescents less likely to comply with treatment overall.

In 2013, Stinson and colleagues aimed to explore treatment service needs of young adults (i.e., ages 18–29) with chronic pain by qualitatively interviewing them and their health care providers [45]. Interestingly, some participants indicated that they place a lower priority on pain management and treatment compared with other demands on their time. Health care providers described a difficulty of maintaining a consistent pain management regimen with this age group due to developmental-related barriers (e.g., difficulty prioritizing healing or reduction of pain at the expense of balancing other areas of life). In fact, one health care provider stated, “An overriding theme for some of our younger patients [is] that, ‘I don’t want to go to CBT, I don’t want to go manage this, I want it gone, I’m 20 years old, I don’t want to have this for the rest of my life” [45]. Translating these results to the present study with a developmentally younger sample, adolescents who are more accepting of pain or believe that they are still able to function despite pain may choose to place more importance on other areas of their life (e.g., school and peers) rather than prioritizing pain management and treatment. They may have focused on independently managing pain and utilizing positive social support (i.e., with encouraging parental responses) rather than relying on psychological treatment. Nevertheless, research is also needed to better understand the role of other potentially relevant barriers for adolescents or families (e.g., stigma) in predicting treatment compliance [45,46].

Findings related to co-occurring functional impairment were significant in expected directions, consistent with initial hypotheses. Similar to previous literature, findings from the present study revealed that lower levels of pain acceptance were associated with increased adolescent-reported functional disability. In addition, lower levels of parental encouragement/monitoring and higher levels of parental protectiveness were both associated with higher levels of adolescent-reported pain intensity, whereas higher levels of parental protectiveness were associated with pain intensity difference scores. Several indices did not evidence significant relations despite having a documented influence in the extant literature with a broader age sample (e.g., self-reported catastrophizing of pain symptoms). In turn, further investigation is warranted to assess what constitutes clinically meaningful indices of functional impairment among adolescents with chronic pain. It may be possible that traditional approaches to assessing functional impairment among pediatric chronic pain (i.e., functional disability, pain intensity, and school absences) may not be sufficient and/or developmentally relevant when considering the experience of adolescents in particular.

The present study has several methodological strengths. A primary strength was the fact that this study was conducted within an active interdisciplinary pediatric chronic pain clinic, which provides translational implications for clinical practice in a real-life setting. The study intentionally focused on youth-reported anxiety and functional impairment because evidence suggests that youth in chronic pain samples are generally better informants of their own symptoms relative to parent report [24,47]. Data were also collected through comprehensive chart reviews of treatment compliance, which provided an objective assessment method in a clinical research setting that attenuated typical concerns related to using self-report indices. In addition, this study uniquely sought to investigate how adolescent resilience strategies (i.e., high level of acceptance, self-efficacy, adaptive caregiver responses to symptoms) elevate their personal commitment to engaging in treatment, though resilience is rarely assessed when considering implications for treatment compliance.

Despite these strengths, the contributions of this investigation must be interpreted within the limitations of the study design and challenges encountered during study implementation. The lack of a formal measurement of treatment compliance or fidelity to the treatment protocol is one such limitation. Comprehensive chart reviews were conducted to measure treatment compliance by assessing treatment sessions missed and/or attended, as well as recording self-reported use and/or engagement in recommendations to the clinician at each appointment. However, this methodological approach may have failed to account for other potential barriers to treatment attendance (e.g., insurance, access to transportation, missing school and/or work and child care), which, in turn, would have impacted engagement in recommendations and treatment compliance as a whole. Overall, chart reviews provided adequate details about participants completing homework assignments and their perceived benefit from skills that were introduced in session. However, the actual fidelity to use these skills, the level of confidence to use these skills at the end of treatment, and continued use of skills following discharge were never concretely measured during the course of the study. Finally, although FIML estimations and weighted summary scores were conducted to statistically account for missing data, some clinically meaningful indices could not be collected at certain time points. Despite significant efforts to obtain follow-up data (i.e., visiting families at other clinic visits, mailing questionnaires home, and calling families to complete questionnaires via phone), most follow-up questionnaires were either not returned or incomplete. Such follow-up assessments would have provided clinically meaningful information about potential changes in adolescent-reported factors over the course of treatment, as well as treatment outcome data.

Keeping the limitations of the study in mind, specific recommendations can be made for future research. First, future studies should incorporate formal measures of fidelity and compliance to the treatment protocol. Treatment protocol checklists should be completed after each session to ensure that the recommendations of that week were effectively followed (using rating scales to identify frequency of use during the past week, confidence with independently using skills, etc.) and a qualitative interview should be conducted regularly to assess feasibility, engagement, and perceived benefit from skills taught, as well as patient outcomes. In addition, future studies should employ larger sample sizes and continue to make efforts towards consistently following up with participants throughout the course of treatment, while taking into account the unique barriers to conducting psychosocial research in a clinic setting. Adequate staffing to help with completing questionnaires efficiently and thoroughly, as well as open communication and collaboration with other medical appointments will help ensure consistent and reliable completion of study measures and follow up assessments. Achieving these goals would help better understand the clinical significance of these risk and resilience factors in predicting treatment compliance and more robust outcomes.

Lastly, expanding the investigation beyond the adolescent context, such as through the inclusion of family, peer, school, and neighborhood factors [9,48], may further enrich unique patterns of risk and resilience impacting adolescents from a broader perspective. Research has suggested that certain barriers to care at both the healthcare and societal level, including a lack of understanding, empathy, and social acceptance (i.e., a child with chronic pain hearing that it was “all in their head”), may deter some adolescents from pursuing treatment and these barriers should be further investigated at this developmental stage [18]. It would be crucial for future research to investigate how factors that influence treatment compliance potentially differ across various pediatric chronic pain conditions (e.g., functional abdominal pain vs. chronic migraine). Given the varying prevalence rates of chronic pain conditions among youth [3], it is important to consider the unique contributions of individual, familial, and psychosocial risk and resilience factors that may play a role in promoting treatment compliance and decreased functional impairment across these different populations from a developmental perspective.

## 5. Conclusions

In conclusion, results from this study revealed that most adolescent risk and resilience factors assessed did not significantly predict improved treatment compliance among adolescents with chronic pain. However, several findings indicated that adolescent resilience factors in fact predict lower levels of treatment compliance. Several adolescent-reported risk factors were associated with increased functional impairment among this sample. Study limitations and statistical concerns warrant that these findings be interpreted with caution. It is important for future studies to devise better strategies of providing consistent follow up within a multidisciplinary clinic setting in order to monitor potential changes in resilience factors through the course of treatment and also to assess clinically meaningful indices of functional impairment for adolescents.

Findings from the present study contribute to our understanding of the unique importance of risk and resilience factors within the developmental context of adolescence, while also highlighting the need for further investigation of other relevant influences towards treatment compliance and functional impairment. Adolescents are in a unique developmental stage marked by neurobiological, psychological, behavioral, and social changes. It is important to identify developmentally specific risk and resilience factors that influence treatment compliance and functional impairment among adolescents in order to optimize assessment and treatment approaches with this population.

From a clinical perspective, these findings support the practical importance of assessing the clinically complex profile of individual, familial, and psychosocial factors among adolescents with chronic pain as part of the treatment planning process. Delineating such characteristics can inform continuing assessment, as well as tailoring treatment targets, recommendations, and outcomes among adolescents with chronic pain within a multidisciplinary treatment setting. Further work is needed to understand how to adapt chronic pain interventions to target specific adolescent needs. Although substantial research is still required to better understand the impact of chronic pain during this developmental period, the present study takes an important step towards highlighting factors related to treatment compliance and functional impairment, particularly from a risk and resilience standpoint.

## Figures and Tables

**Table 1:** Bivariate Correlations among Continuous Study Variables.

Variable	1	2	3	4	5	6	7	8	9	10	11	12	13	14	15	16
1. age																
2. PROMIS YA	0.08															
3. ARCS-A Enc/Mon	−0.17	0.08														
4. ARCS-A Prot	−0.33	0.20	0.61 ***													
5. PCS-C	−0.24	0.33 *	0.07	0.27 *												
6. PSES	−0.14	0.27	0.06	0.29 *	0.51 ***											
7. T2missed	0.12	−0.04	0.00	0.11	−0.03	0.22										
8. T2attend	0.05	0.05	−0.11	0.05	0.07	0.01	0.04									
9. T2txrecs	0.06	−0.09	−0.30	−0.16	−0.13	−0.14	0.22	0.56 ***								
10. T3missed	0.12	0.00	0.03	0.16	0.03	0.22	0.94 ***	0.18	0.263 *							
11. T3attend	0.00	0.09	−0.15	0.04	0.09	0.04	0.09	0.96 ***	0.52 ***	0.25 *						
12. T3txrecs	−0.06	−0.04	−0.18	−0.07	−0.14	−0.01	0.33 **	0.55 ***	0.60 ***	0.41 ***	0.63 ***					
13. painhigh	−0.02	0.21	−0.16	0.24	0.22	0.32 *	0.03	0.30 *	0.23	0.04	0.28	0.24				
14. paindiff	−0.03	−0.06	−0.03	0.14	−0.17	0.02	0.378 *	0.28	0.27	0.418 **	0.29	0.314 *	0.19			
15. PROMIS YPI	0.03	0.39 **	−0.31	−0.18	0.20	0.350 *	−0.03	0.10	0.00	0.02	0.12	0.09	0.31	0.00		
16. CPAQ	0.15	−0.38 *	−0.12	−0.24	−0.29	−0.55 ***	−0.03	−0.14	−0.12	−0.1	−0.19	−0.14	−0.25	0.16	−0.44 **	
M	15.00	12.61	20.75	25.42	27.60	20.29	1.39	3.00	0.49	1.78	3.80	0.61	8.50	5.95	14.51	39.12
SD	1.68	8.73	5.97	10.41	12.38	6.05	1.85	3.14	0.43	2.26	4.14	0.67	1.50	2.00	4.90	10.85
*n*	64	49	57	57	58	58	64	64	64	64	64	64	40	47	47	46

Note. PCS-C: Pain Catastrophizing Scale-Child; PSES: Pain Self-Efficacy Scale; CPAQ: The Chronic Pain Acceptance Questionnaire; ARCS: Adult’s Responses to Child’s Symptoms; PROMIS YA: Youth Anxiety Short Form; PROMIS YPI: Youth Pain Interference Short Form; T2 = Time 2 (3 months post intake); T3 = Time 3 (6 months post intake); Enc/Mon = Encouragement/Monitoring; Prot = Protectiveness; missed = total missed (no show and cancel) treatment sessions; attend = total treatment sessions attended; tx recs = adolescent-reported use and/or with treatment recommendations; painhigh = highest/worst pain. intensity rating at T1; paindiff = pain intensity difference rating at T1. * *p* < 0.05; ** *p* < 0.01.; *** *p* < 0.001.

**Table 2:** Multiple Regression Analyses with Time 1 Adolescent-Reported CPAQ, ARCS-A, PSC-C, and PSES Indices Predicting Time 2 and Time 3 Treatment Compliance Variables.

	**Time 2 (3 months; *n* = 62)**		
Time 1 Predictors	3mo missed	3mo attend	3mo tx recs
CPAQ	0.21	−0.33	−0.42 *
ARCS-A Enc/Mon	−0.06	−0.25	−0.41 *
ARCS-A Prot	0.12	0.19	0.14
PCS-C	−0.22	0.04	−0.11
PSES	0.43 *	−0.22	−0.31
	**Time 3 (6 months; *n* = 61)**		
	6mo missed	6mo attend	6mo tx recs
CPAQ	0.17	−0.35 *	−0.14
ARCS-A Enc/Mon	−0.16	−0.27	−0.17
ARCS-A Prot	0.33	0.18	0.07
PCS-C	−0.31	0.03	−0.13
PSES	0.74	−0.21	−0.02

Note. Coefficients are standardized Beta weights. Analyses considered prediction from Time 1 CPAQ, ARCS-A, PCS-C, and PSES indices controlling for Time 1 adolescent-reported anxiety symptoms on the PROMIS-YA. Enc/Mon = Encouragement/Monitoring; Prot = Protectiveness; missed = total missed (no show and cancel) treatment sessions; attend = total treatment sessions attended; tx recs = adolescent-reported use and/or engagement with treatment recommendations. * *p* < 0.05.

**Table 3:** Multiple Regression Analyses with Time 1 Adolescent-Reported CPAQ, ARCS-A, PCS-C, and PSES Associations with Time 1 Functional Impairment Variables.

	Time 1 Impairment Variables (*N* = 64)		
Time 1 Predictors	Interference	Pain High	Pain Diff
CPAQ	−0.34 *	−0.06	0.22
ARCS-A Enc/Mon	−0.27	−0.42 **	−0.24
ARCS-A Prot	−0.15	0.41 *	0.38 *
PCS-C	0.02	0.00	−0.26
PSES	0.15	0.13	0.14

Note: Coefficients are standardized Beta weights. Analyses considered prediction from Time 1 FES variables while controlling for Time 1 adolescent-reported anxiety symptoms on the PROMIS-YA. Enc/Mon = Encouragement/Monitoring; Prot = Protectiveness; Interference = functional disability on the PROMIS-YPI; Pain high = highest/worst pain intensity rating; Pain diff = pain intensity difference rating. * *p* < 0.05; ** *p* < 0.01.
